# Atomistic Simulations of Functionalized Nano-Materials for Biosensors Applications

**DOI:** 10.3390/ijms23031484

**Published:** 2022-01-27

**Authors:** Sutapa Dutta, Stefano Corni, Giorgia Brancolini

**Affiliations:** 1 Department of Chemical Sciences, University of Padova, Via Marzolo 1, 35131 Padova, Italy; sutapa.dutta@unipd.it (S.D.); stefano.corni@unipd.it (S.C.); 2 Institute of Nanoscience, CNR-NANO S3, Via G. Campi 213/A, 41125 Modena, Italy

**Keywords:** biosensors, atomistic simulations, surface functionalization, proteins

## Abstract

Nanoscale biosensors, a highly promising technique in clinical analysis, can provide sensitive yet label-free detection of biomolecules. The spatial and chemical specificity of the surface coverage, the proper immobilization of the bioreceptor as well as the underlying interfacial phenomena are crucial elements for optimizing the performance of a biosensor. Due to experimental limitations at the microscopic level, integrated cross-disciplinary approaches that combine in silico design with experimental measurements have the potential to present a powerful new paradigm that tackles the issue of developing novel biosensors. In some cases, computational studies can be seen as alternative approaches to assess the microscopic working mechanisms of biosensors. Nonetheless, the complex architecture of a biosensor, associated with the collective contribution from “substrate–receptor–analyte” conjugate in a solvent, often requires extensive atomistic simulations and systems of prohibitive size which need to be addressed. In silico studies of functionalized surfaces also require ad hoc force field parameterization, as existing force fields for biomolecules are usually unable to correctly describe the biomolecule/surface interface. Thus, the computational studies in this field are limited to date. In this review, we aim to introduce fundamental principles that govern the absorption of biomolecules onto functionalized nanomaterials and to report state-of-the-art computational strategies to rationally design nanoscale biosensors. A detailed account of available in silico strategies used to drive and/or optimize the synthesis of functionalized nanomaterials for biosensing will be presented. The insights will not only stimulate the field to rationally design functionalized nanomaterials with improved biosensing performance but also foster research on the required functionalization to improve biomolecule–surface complex formation as a whole.

## 1. Introduction

Biosensors [1,2] can detect finite concentrations of biological substances (analyte) in bodily fluids by producing response signals. Today, due to this specific mechanism, biosensors are ubiquitously studied not only in academia but also in industries for applications such as food safety, environmental monitoring, bio-analysis, and detection of biomarkers associated with biomedical diagnostics [1,3,4,5].

Typically, a biosensor consists of five different components; analyte, bioreceptor, transducer, electronics, and display; the focus here will be on the former three of these components, and their interfaces. One of the vital characteristics [2,6] of a good biosensor is selectivity, i.e., the sensor’s bioreceptor molecule should specifically recognize the desired analyte in presence of all other contaminants. Thus, the selection of a suitable bioreceptor for the bio-recognition phenomenon is the criterion one needs to keep in mind while developing a biosensor. In addition, choosing the proper immobilization method for entrapment of the bioreceptor in the vicinity of the transducer is also required. The second key factor is sensitivity; an ideal biosensor must detect minute (~ng/mL or ~fg/mL) concentrations of analyte from the test sample. The lowest detectable quantity of the analyte to produce a measurable signal is known as the limit of detection (LOD) and the range of concentration for which the performance of the sensor is scientifically relevant is known as the linearity of the sensor. The successful design of a biosensor, therefore, places emphasis on the enhancement of linearity and betterment in LOD. Strategies combining in silico modeling with laboratory experiments are emerging as a new paradigm to tackle the issue of developing novel diagnostic tools [7] to detect a broad spectrum of biomarkers. The large amounts of data which can be collected, tested, and pre-screened from in silico design simulations enable fast recognition of functionalized nanomaterials able to detect specific biomarkers. In turn, the time of the first phase of experimental studies is reduced.

However, correctly fulfilling all the criteria for a novel biosensor from experiments can be challenging [8,9]. We refer specifically to: sample matrix effect, the complicated procedure of surface functionalization of the nanomaterial creating detectable signals and sample preparation steps, the bulky architecture of the system, amplification in output response signals for real-time detection, critical data analysis technique, and careful control of physicochemical conditions. These are all challenges that one needs to overcome to integrate a biosensor for real-life applications. Hence, as a complementary approach, scientists have started addressing the domain of biosensors from a computational perspective.

Considering the complexity of the bio–nano interface, one must question to what extent theory and simulations can be used to study these systems in a realistic, meaningful way. In this review, we will provide an overview of the properties of functionalized nanosurfaces that can be captured within different levels of theoretical description.

In particular, we highlight the contribution of microscopic simulations (from atomistic to coarse-grained) to the design of the analyte binding region of the sensor, the key element for sensitivity and selectivity. We also focus on the specific parameters which need to be addressed through simulations and how it is possible to correlate those to the corresponding physical characteristics of a sensor, accompanied by a short description of the system and the computational techniques. In addition, some of these parameters, such as surface morphology on atomistic level or role of biomolecular–electrolyte dynamics particularly at high ionic strength, in modulating the response of a potentiometric sensor by distinguishing changes in surface potential occurring on ~µs range, are conventionally neglected [10] in experiments and thus can be understood only through simulation. However, due to the involvement of a huge number of atoms in every different component of a biosensor, the collective effect of the transducer surface, chemical moieties as the bioreceptor, and the analyte embedded in a solution, can be quite difficult to decode even from simulations. Thus, our review has the goal of presenting the state-of-the-art and inspiring the development of further strategies to substantiate future accomplishments.

More specifically, in this review, we will focus on the design of functionalized nanomaterials for biosensors’ fabrication, where the biorecognition elements (BREs) are conjugated by functionalizing the surface of the nanomaterials. The usage of nanomaterials in biosensing applications is one of the fruitful strategies [11,12,13,14,15,16] in the broad spectrum of state-of-the-art sensor developments. These particular materials with ~nm dimension can optimize LOD, selectivity, sensitivity along with response time of a biosensor, primarily [11,12,13,14,15,16] because of the high surface to volume ratio, high electrical conductivity, catalytic activity accompanied by enhanced magnetic properties. Nanomaterial functionalized with diverse inorganic and organic moieties and synthesis of doped nanocomposite can contribute to biocompatibility and flexibility in this research field. They can be co-immobilized with the receptor and, at the same time, can be integrated as a sensitive surface of the transducer. Moreover, the miniaturization of the entire complex entity into lab-on-a-chip devices with point-of-care detection facilities has made nanosensors the next-generation biosensor.

More specifically, we are reviewing atomistic simulations involving the interaction between proteins to be detected and functionalized surfaces, with the aim of mimicking the biosensor. The complexity associated with size, shape, chemical composition, the molecular structure of the various surface, specific adsorption of proteins over different surfaces, choice and suitable interaction potential, and transferability of force field involving protein–surface interaction makes this regime challenging and elusive both from theory and experimental perspectives [17,18].

Recent computational strategies have been applied to design surface functionalization of nanomaterial-based nanosensors, namely (i) two-dimensional carbon-based substrate nanomaterials [19,20,21] (e.g., graphene) and (ii) self-assembled monolayer gold nanoparticles (AuNP) [22,23,24], but also to the description of organic-based biosensors which rely on (iii) polymers [25,26,27,28], such as poly (acetylene), poly (3,4-ethylenedioxythiophene) (PEDOT), poly (thiophene) (PTh), poly (p-phenylene vinylene) (PPV), poly (pyrrole) (PPy) and poly (aniline) (PANI), as used in electrochemical biosensors and finally of biomolecule based nanosensors, such as (iv) aptasensors [29,30,31] or (v) nano-pores [32,33].

## 2. Graphene-Based Biosensors

Graphene and graphene oxide (GO) are two-dimensional lattices of sp^2^ bonded carbon in a honeycomb structure proven to a play crucial role [34,35,36,37,38] in the detection of biomolecules, such as glucose, cholesterol, NADH, DNA, RNA, and many more. Graphene possesses high electrical conductivity due to π electrons while GO includes structural defects which facilitate ease of functionalization for electrochemical applications [2] toward the desired biomolecule. In both cases, the strong adsorption of the biomolecule on the substrate occurs owing to large regions with delocalized π electrons [35]. In addition, the inherent structural defects [36] of the lattice enhance the electron transfer rate between the biomolecules and the graphene derivative transducer, leading to excellent electrocatalytic activity. Furthermore, the large specific surface area (2630 m^2^/g for single-layer graphene) contributes to high sensitivity [37], followed by miniaturization of the complex architecture. The graphene monolayer also exhibits high optical transparency [39] and graphene oxide demonstrates a fluorescence quenching effect [39], making it suitable for optical readout. These features make graphene derivative materials potential candidates in the field of the design of novel biosensors.

In a recent study [40], a graphene-based fluorescence biosensor was investigated for label-free detection of ssDNA and dsDNA based on aggregation-induced emission molecules (AIE) and complementary DNA (comDNA) adsorbed on GO. The sensing mechanisms rely on the capability of turning the AIE fluorescence on/off by adjusting its distance from the GO sheet. Namely, with GOs, it was possible to quench the emission from AIE-ssDNA but further addition of comDNA could recover the emission, as ssDNA can hybridize with its complementary ssDNA. The advantage of the designed sensor with respect to conventional fluorophore dyes is that it does not require to be covalently tagged on DNA, since covalency often leads to self-quenching.

Remarkably, the design of the sensor was supported by computational modeling, which not only allowed the rationalization of the GO-DNA interactions but also demonstrated that the ssDNA had a higher binding affinity with respect to dsDNA. Classical molecular dynamics (MD) simulations were performed on flat and rigid graphene nanosheets (GNS) along with the single (ssDNA) and double-stranded (dsDNA) DNA [40]. Carbon atoms were treated as neutral Lennard Jones (LJ) particles and parameters were implemented within a CHARMM27 force field [41]. The fluctuations of the number of contacts between DNAs and GNS along with the variation of the center of mass distances between the DNAs and the surface provided evidence that the ssDNA undergo stronger interactions towards GNS with respect to dsDNA. The comparatively weaker adsorption of dsDNA on GNS was further supported by the preservation of the duplex throughout the entire simulation and by the moderate fluctuation of the radius of gyration of dsDNA. Finally, the calculated binding affinity revealed that for ssDNA, the underlying π–π interaction was stable, whereas, for dsDNA, the orientation was metastable since the orientation of aromatic rings of purines and pyrimidines over GNS were not maintained as stable during the simulations. The detailed understanding of the interaction between DNA and GNS from simulations supported the capability of this graphene-based biosensor to detect sequence-specific DNA.

Recent computational work on graphene-based biosensors was performed by Pang et al. [42] using multiscale simulations to probe the role of temperature fluctuations and material thickness on the performance of a graphene/Au-based surface plasmon resonance (SPR) sensor. The structure and the dielectric constant of two types of Au lattice (cubic and triclinic), followed by monolayer and multilayer graphene structures with AA-stacking and AB-stacking, were optimized using density functional theory (DFT). Next, the embedded atom model (EAM) [43] potential, namely an extensively studied empirical method used to describe pair interactions from a consistent set of embedded functions which are fitted to experimental observations (e.g., activation energies, equilibrium lattice constants, elastic constants, surface sublimation as well as vacancy-formation energies), was used to include the contribution from the Au atoms on MD simulation. From MD simulations, the thermal expansion coefficient was derived yielding to the photothermal effect which is known to affect the performance of the SPR biosensor. The biosensor sensitivity was shown to be influenced not only by the lattice type but also by the thickness of the graphene’s interfacial layers. Similarly, from first principle calculations, it was possible to observe that the dielectric constants of the Au–graphene sensor have a significant dependence on temperature. Thus, within a theoretical framework, it was possible to conclude that the detection sensitivity of this type of sensor can be optimized by modifying observables, such as the dielectric constant and the thickness of the components at different temperatures.

**Figure 1:**
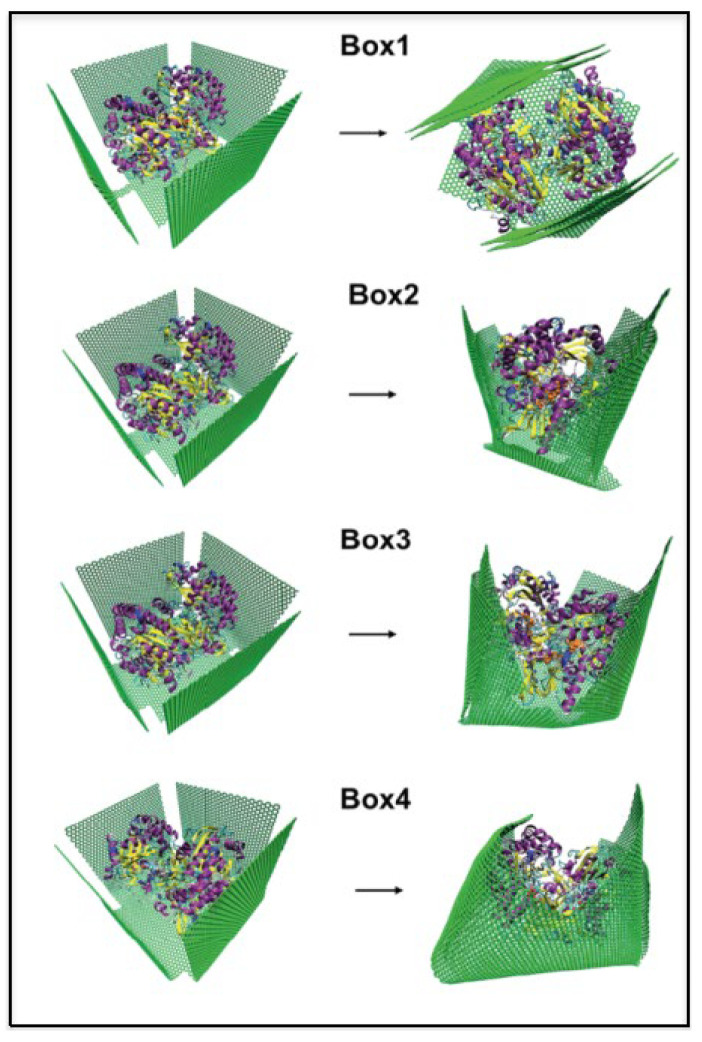
Different orientations of graphene sheets connecting via different widths of linkers, encapsulating the glycoprotein, are observed to affect the catalytic activity of the enzyme as well as the protein detection mechanism by the graphene-based sensor. Reprinted with permission from Ref. [45]. Copyright© 2022 Royal Society of Chemistry.

Similarly, the detection of glucose in blood plasma was found [44] to be related to the detection of the glycoprotein “glucose oxidase” in the presence of graphene. Glucose oxidase is an enzyme that has a strong specificity towards glucose owing to the size and shape complementary to its active site. As a result, it has become a benchmark system in electrochemical-based biosensors, where detection of glucose in the presence of this enzyme can be monitored by tracking a change in the output signal. A theoretical study [45] probed the role of different shapes and orientations of graphene in modulating its capability to interact with the enzyme (Figure 1). The study was performed using an OPLS [46] force field, in which carbon atoms of graphene were considered neutral and the non-bonded parameters for naphthalene were used for graphene. Initial geometry optimization of the graphene sheet was performed at the DFT level. Next, classical MD simulation of glucose oxidase in the presence of a catalytic cofactor, named flavin adenine dinucleotide, was carried out under different environments: (i) a single graphene sheet, (ii) multiple unconnected graphene sheets stacked in a flat conformation, and (iii) sheets connected via linkers of different chain lengths forming a flower-like shape. Several microscopic parameters were addressed from the simulation trajectories, e.g., the surface contact area and center-to-center distance between two monomeric units of the protein (which is a dimer), the contact area between graphene and the protein, and the distance between protein and the cofactor. Simulations revealed that graphene sheets exhibited the maximum encapsulation effect in the flower-like shape. As a result, the compact secondary structure of the dimeric protein was disrupted. The cofactors, as well as the active residues, moved away from the biologically relevant orientations and the catalytic activity of the protein was damaged. Results highlighted the fact that even if properly immobilized in presence of the graphene sheets, the protein will be perturbed and its capability of indirectly measuring glucose from a blood sample would be damaged. The interaction energies between the graphene sheet and the charged amino acids were calculated through the DFT method. We envisage that the insights provided by this study would provide additional knowledge in view of developing novel graphene–enzyme biosensors [45].

**Figure 2:**
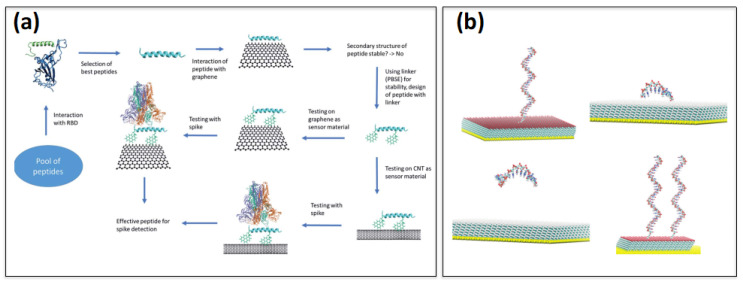
(**a**) In silico modeling pipeline for the peptide design and testing for its usage as a graphene (or CNT)-based biosensor for SARS-CoV-2. Reprinted from Ref. [47]. (**b**) Different initial orientations and different densities of ssDNA attached on SAM are used to optimize the performance of a nucleic acid-based biosensor. Reprinted with permission from Ref. [48]. Copyright© 2022 Royal Society of Chemistry.

Recently [47], a biosensing platform based on an in silico modeling pipeline (Figure 2a) was developed to test the effectiveness of graphene sheets and carbon nanotubes (CNT), to act as a sensor-support structure for the detection of SARS-CoV-2. The authors screened twelve different peptides based on their potential binding affinity toward the receptor-binding domain (RBD) of the spike protein of the virus, using a series of atomistic molecular dynamic simulations performed with CHARMM36 FF [41]. Two over twelve peptides were selected and explored for their potential use in biosensing applications for SARS-CoV-2, namely, they were used to functionalize the nanomaterials, both graphene and CNT, upon addition of a proper linker. Based on the in silico computed interaction between the substrate, peptide, and spike protein, it was possible to evaluate the potential for the screened peptides for the detection of the spike protein of the virus.

To summarize, by means of atomistic simulation it was possible to disclose [40] the microscopic interaction between DNA and graphene surfaces and to predict the sequence-specific detection capability of graphene over DNA strands. Similarly [42], the photothermal effect in biosensors could be described through multiscale simulations, and in the case of graphene/Au based sensors, the potential role of interfacial surface layers and the dielectric constant was revealed using quantum calculations. Multiscale simulations have shed light on the effect of different shapes and conformation of graphene sheets in the presence of different concentrations of linkers encapsulating a protein involved in catalysis [45], also providing an estimate of the protein–surface interaction energy at the quantum level. Finally, in a recent work, an in silico workflow was applied to assist the design of biosensors for the detection of SARS-CoV-2.

### Self-Assembled Monolayer-Based Biosensors

Self-assembled monolayer (SAM) [49] plays a pivotal role in the definition of biosensor devices, specifically because of its miniature size and versatility in designing [50] immobilization matrix of biomolecules. These types of chemical moieties can spontaneously self-assemble [49,50,51,52,53] into complex architecture under optimal conditions, once attached to the substrate of the biosensor. Thus, one can achieve molecular level of precision over the SAM density, thickness, chain length, charge accumulation, orientation as well as arrangement pattern of the single units at the interface. SAM enhances sensitive detection of biomolecules. In this context, gold nanoparticles are routinely used [22,23,24] as substrate owing to optical, electronic properties as well as for biocompatibility, large surface area, and easiness of production and surface modification. In particular, collective oscillation of electrons in the conduction band can occur upon irradiation of a gold surface with a particular wavelength (and with proper illumination geometries to satisfy light momentum conservation), leading to the so-called surface plasmon resonance (SPR). Thus, the detection of changes in the oscillation frequency of the surface upon biorecognition can be exploited in the design of a biosensor. SAM on gold can also be used in electrochemical biosensors, [54,55] where the electrons can be transferred from the gold electrode to the redox center of the immobilized biomolecule.

Simulations have been used to optimize the performance of a SAM-based nucleic acid biosensor [48], where the thiol head group of an alkane chain adhered to a gold substrate, and the terminal group was used to immobilize ssDNA. Alkane chains with four different functional charged groups along with two different chain lengths were considered. All-atom MD simulations were prescribed to model DNA using the AMBER FF14SB [56] force field and AMBER GAFF [57] for a SAM attached to a gold surface. Other systems with different initial positions of ssDNA with respect to the surface, were studied, using different numbers of DNA base pairs and different numbers of ssDNA strands at varying distances from the substrate (Figure 2b). The binding interaction energy and the distance between DNA and SAM were collected over the simulated trajectories. The distance between DNA and ions, ions and ssDNA was also addressed to understand the potential role of ions in modulating adsorption of ssDNA over SAM. The radius of gyration and the diffusion dynamics of ssDNA on the surface were also computed. Results emphasized the strong contribution coming from both ions and the hydration layer during the adsorption and the diffusion of DNA on top of the surface, particularly in the presence of a charged substrate. Principle component analysis revealed the mobility of DNA and the preferred initial orientation associated with the maximum absorption, directly related to the sensor performance.

**Figure 3:**
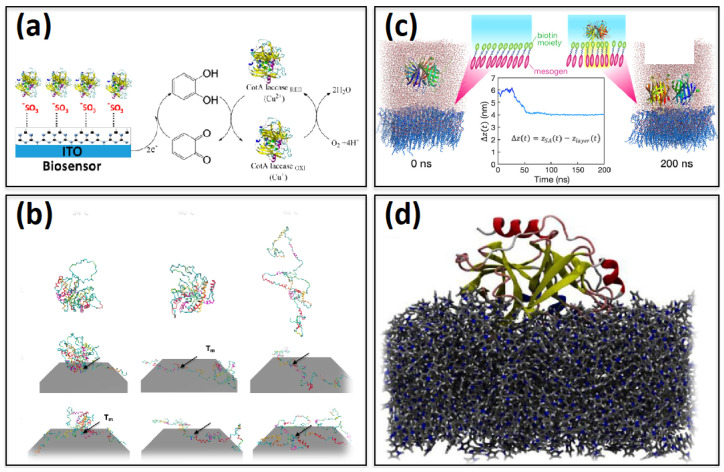
(**a**) ET rates of CotA laccases improved by immobilizing it on sulfonic group-modified graphitic carbon nitride (Sg-CN). Reprinted with permission from Ref. [58]. Copyright© 2022, American Chemical Society. (**b**) Thermal unfolding of β-Gal during simulation at different temperatures corresponding to different initial attachment points for each surface-tethered enzyme. Reprinted with permission from Ref. [62]. Copyright© 2022, American Chemical Society. (**c**) Difference in the detection of streptavidin by monolayers with biotins and by biotin-free amphiphilic mesogenic molecules. Reprinted with permission from Ref. [63]. Copyright© 2022, American Chemical Society. (**d**) A snapshot of PSA protein embedded in MIP polymer as studied for a polymer-based biosensor, figure taken from Ref. [64] with permission from © 2022 Elsevier.

Recently [58], Puente-Santiago et al. reported an amperometric biosensor with a significantly higher electron transfer rate based on a recombinant bacterial CotA laccase enzyme, adhered on a sulfonated graphitic carbon nitride (Sg-CN) surface (Figure 3a). Atomic charges of the Sg-CN surface were obtained through the restrained electrostatic potential (RESP) method. First, the preferred orientation of the enzyme over the substrate was revealed through parallel tempering Monte Carlo simulations [59,60]. Next, an all-atom MD simulation was performed for the entire system using the CHARMM36 force field [41] in the GROMACS [61] package. The residues coming in close proximity to the surface were addressed over the simulated trajectory. Interestingly, it was observed that this protein behaved as a Janus particle with a specific distribution of positive and negative charged patches over its surface, whereas the Sg-CN surface became hydrophilic in nature due to adjacent S atoms of the hydrophobic g-CN layer. Thus, in the presence of this self-assembled surface, a strong protein–surface electrostatic interaction could be mediated through the basic residues of the metalloenzyme protein. As a result, the protein–surface absorption and the electron transfer rate were enhanced, compared with the free protein. The study provided the understanding of how to optimize the electrocatalytic activity of a genetically engineered metalloenzyme by proper surface functionalization in view of biosensor applications.

Another example of a SAM-based biosensor was reported [62], namely, a β-Gal enzyme on an (ethylene glycol)_4_-maleimide-terminated self-assembled monolayer. In one case, this enzyme was tethered to the surface via a rigid α helix, while in another configuration it was attached with a comparatively flexible loop as shown in Figure 3b. The Karanicolas and Brooks’ structure-centric (Go) protein model [65,66] was employed in this work, in which C_α_ atoms are considered as representative of each residue and only hydrogen bonding is incorporated in defining native contacts. The interaction between protein and surface was further investigated [67] by proper parameterization of this coarse-grained (CG) model. More specifically, the potential function was made of a repulsion term, an adsorption well, a desolvation term, and hydrophobic contribution between hydrophobic residues and the self-assembled layers. The covalent bond between the cysteine residue and thiol was incorporated through harmonic potential. Next, the thermal stability of this enzyme for different tethering sites was investigated with replica exchange molecular dynamics (REMD) method [68,69]. Simulation data suggested that although the activity of this enzyme did not depend on the tethering site, thermal stability was indeed affected. For the loop adhered system, the protein underwent a prominent interaction with the surface which gradually lead to a thermal unfolding. Thus, from this work, it was deduced that the present computational protocol can be systematically applied to vary the tethering sites of the enzyme over the substrate, with the aim of identifying those that can potentially enhance the thermal stability of surface-supported biocatalysts.

A hybrid photonic-plasmonic antenna nanocavity-based biosensor associated with a single molecule was proposed [70] by Liang et al. More specifically, the immobilization of a xeroderma pigmentosum (XP) gene-encoded protein XPA in the presence of a DNA sequence was reported on a self-assembled monolayer of alkane thiol, named 11-mercaptoundecanoic acid (11-MUA), tethered on a gold surface. This particular type of biosensor did not require traditional fluorophores labeling, being fluorescent photobleaching and with a resolution time of ~millisecond. Thus, the proposed system had the potential to perform as an enhanced fluorescent-based sensor with respect to the existing biosensors. In order to probe the XPA-DNA interaction, a mismatched DNA sequence was considered different with respect to the specific one. To support the choice, MD simulations were performed and the solvent-accessible surface potential for both the normal and the mismatched sequences were evaluated. Results reported a higher value of surface potential for the mismatched DNA sequence, accompanied by an abnormal twist in its conformation in the presence of XPA, thus justifying the sequence-specific sensitive detection of DNA of the device. Furthermore, a geometry optimization of gold (100) surface attached to the Lysine residue of XPA, bound to 11-MUA and the free Lysine-MUA complex, was optimized at the DFT level. The partial charge density calculations extracted from the ab initio simulations suggested that MUA was acting as an insulator, hindering the charge transfer from gold to protein. Therefore, simulations were applied to support the use of the self-assembled MUA to preserve the native properties of a protein in designing the biosensor.

Two different molecular dynamics simulation studies [63] on a monolayer of mesogenic (liquid crystal) molecules (Figure 3c) interacting with Streptavidin, a tetrameric protein [71], have been conducted. In one case, the mesogenic molecules had already adhered to the biotin bioreceptor, whereas, in another system, the mesogenic layer was simulated without the biotins. The two different systems were simulated in water in GROMACS [61] using an Amber ff99SB-ILDN and GAFF forcefield [56,57]. RESP charges [72] were derived at the quantum level with GAUSSIAN09 [73]. In addition, a system composed of the biotinylated monolayer in the presence of a streptavidin–biotin complex was also taken into account. The distance between the streptavidin and the surface along the *z*-axis was recorded as a function of the simulation time to understand the degree of adsorption of the streptavidin on top of different surfaces. The results reported that without biotin, streptavidin required a longer time scale to adhere to the surface. In addition, the diffusion behaviors of the mesogenic molecules, contributing to the binding, remained constant over time with respect to the surface of mesogenic molecules without biotin. The stronger the binding, the lesser the diffusion coefficient of interfacial water on the surface. Binding energetics were computed in terms of Columbic and Van der Waals terms, justifying a higher binding affinity for the biotinylated mesogenic surface. The strongest binding induced an increased orientational order parameter and molecular tilting of mesogenic molecules in terms of the normal of the surface. Simulations could assist the evaluation of all of these microscopic parameters which have been shown to be relevant to developing optimal biosensors based on a liquid crystal–aqueous interface.

In a recent work [74], the immobilization of the biotin bioreceptor linked to the polyethylene glycol (bPEG) and grafted to the gold surface was simulated for the design of the electrode of a biosensor for the detection of Streptavidin. The computational study disclosed the effects of two different surface coverage densities of biotin along with their exposure to water, thus different concentrations of bPEG and the ligand biotinylation ratio (bPEG:PEG) were investigated. The interaction between the functionalized gold surface (Au (111)) and the protein has been studied using the well-established GolP Force field [75] compatible with the standard OPLS format. Different microscopic parameters contributing to the ligands’ conformation, were analyzed. Namely, solvent accessible surface area (SASA), distance distribution of biotin over the surface, the radius of gyration of bPEG/PEG linkers were collected and analyzed to identify the optimal ratio between coverage ligand density and degree of biotinylation. It was observed that in the mixed density system, the hydrophilic nature of PEG had the effect of enhancing a conformational transition of spacers from the mushroom-like conformation to the brush-like conformation; as a result, biotins were pushed away from the gold surface leading them to a higher exposure to the solvent. This effect was further enhanced in the case of a higher density coverage of half biotinylated ligands; in this case, the exposure of biotins toward solvent was maximum. The atomistic calculations provided guidelines for the optimization of the performance of a designed biosensor by means of tuning its surface functionalization properties.

To summarize, various computational techniques were applied to investigate SAM surface functionalization: (i) MD simulations [48] were used to reveal optimal physical parameters for the maximum absorption of a target DNA sequence on the basis of RESP quantum atomic charges [58] and on geometry optimization from DFT [70] of the SAM; (ii) the preferred orientations of the biomolecules over the surface [58] were derived from parallel tempering MC simulation and the binding affinity between the biomolecules and the surface was addressed via MD simulation [58,70]; (iii) the replica exchange molecular dynamics method was applied on the coarse-grained (CG) model of enzyme over SAM based biosensor. In all cases, tunable microscopic parameters obtained from MD simulations were used to guide the design of novel biosensors.

## 3. Polymer-Based Biosensors

Polymer-based biosensors rely on conducting functional organic materials [25,26,27,28] which are stable, biocompatible, and easily tunable. These materials possess strength, flexibility, elasticity, and intrinsic electronic, optical, thermoelectric properties owing to delocalized π electrons across the structure. The analyte biomolecules can be entrapped over a polymer substrate via physio-chemo absorption and those systems are mainly used as elements of electrochemical biosensors.

Molecularly imprinting polymers (MIPs) have become a promising approach to select artificial receptors for a given analyte. Realizations of MIP involve three steps: (i) the formation of pre-polymerization complexes between the monomers and the template, (ii) the polymerization, and (iii) the removal of the template.

The tryptophan (W) amino acid is known as a biomarker for neurological activity and neurotoxicity; thus it requires low-cost analytical detection without undergoing degradation [76]. Several molecularly imprinted polymeric (MIP) surface-based sensors using polypyrrole (PPy) oligomer with a varying number of rings were designed and studied by means of atomistic simulations to understand selective and sensitive detection of W. MD simulations with an implicit water model [77] CHARMM force field [41,78] have been performed in NAMD [79] starting from DFT optimized geometries. The dynamic of the underlying interactions between py and indole rings (in terms of hydrogen bonds and dipole-dipole components) was found to be proportional to the increase in the polymer chain length. However, the study [76] suggested that further research work based on more than one chain of the polymer was required in order to disclose the effect of the shape and size of the complementary cavity of MIP to entrap the target molecule, being crucial for the proper fabrication of a biosensor.

In another study [64], an electrochemical biosensor based on the copolymerization of PPy over a gold electrode for the detection of a prostate-specific antigen (PSA) was investigated. For this PPy-PSA-MIP sensor, the ff14SB force field [56] in AMBER was used [80], and the packmol [81] software was applied to pack PSA by PPy molecules in both *cis* and *trans* conformations (i.e., according to the Boltzmann distribution of enthalpy obtained via quantum chemical calculation). In the study, the lock–key mechanism was mimicked by MD simulation, based on the capability of MIP to specifically bind the analyte with which it was jointly templated during the copolymerization process, thus forming a complex. In a second step, template removal was required, in order to obtain a cavity inside the polymer matrix, which was available for a selective rebinding of the analyte. In this case, in the initial configuration, PSA was fully covered by PPy in all directions, thus MIP was not functional as the protein could not escape through a layer of PPy. The next goal was to find the ideal direction to remove the PPy so that the MIP could be active. For removal of the PPy layer, six different systems were chosen and for each dimension, two preferred orientations named “top” and “bottom were assigned to remove PPy. Initially, PPy was placed by ~50 Å from PSA in each corresponding system, these six systems were thus subjected to MD simulations. The preferred direction for the removal of the PSA template from PPy-MIP (Figure 3d) was addressed in terms of the adsorption energy and protein-PPy contact surface area, as extracted from the simulated trajectories. The interfacial (involved) amino acids in terms of hydrogen bond and π interactions, were identified and the simulation results justified the choice of PPy-MIP sensor for the sensitive detection of PSA. The designed sensor was applied to the detection of PSA concentration in human serum and the results were in agreement with those obtained by the standard ELISA method, thus highlighting the contribution of computational studies to the design of the PPy-MIP surface.

A DFT study [82] on a monomeric poly (N-isopropylacrylamide) (PNIPAM) grafted on a (graphene oxide) GO surface and complexed with a single nucleobase was performed, with the aim of designing a switchable cancer diagnostic tool, based on the well-known thermal-responsive property of the NIPAM polymer acting as an “on/off”-switch. A GO model [83,84], including one epoxide, three hydroxyl groups at the basal plane, and a carboxyl group at the border, was chosen. PNIPAM chains were attached to the GO surface through carboxylic conjunctions and the initial nucleobases were placed at 2.5 Å distance above functional groups and relaxed by DFT. After obtaining the initial geometry of each structure and their complex systems in the DMol3 [85] package, the systems were relaxed, and the electronic structure of GO-NIPAM-nucleobase was addressed using a generalized gradient approximation (GGA) with the Perdew–Burke–Ernzerhof (PBE) functional [86]. The adsorption energy of the nucleobases over the GO surface, density of states, vibrational spectra, HOMO-LUMO gap, and electrostatic potential disclosed the most favorable conformations. A single nucleobase adsorbed on the surface of the GO/NIPAM was used to depict the interaction of the aptamer with the nanomaterial. The cancer-specific protein was thus docked on the aptamer using the ZRANK module of Discovery Studio [87], followed by MD refinement of the whole complex, i.e., GO surface, 50 PNIPAM chains, Wy5a aptamer, and the α6β4 protein, using the universal force field (UFF) [88]. To disclose the thermal responsive behavior of the polymer within the PNIPAM-grafted GO surface, the variation of the radius of gyration of PNIPAM and the distance fluctuation between protein and aptamer were evaluated at low (298 K) and high temperature (310.7 K), respectively. Results suggested that at lower temperatures a linear conformation of the polymer hinders the interaction between aptamer–protein, while at higher temperatures, the globular conformation of PNIPAM enhanced the detection mechanism. The study provided a strategy to develop a tunable thermal responsive biosensor.

Boroznjak et al. considered [89] three functional monomers (m-phenylenediamine (mPD), dopamine (DA), 3,4-ethylenedioxythiophene (EDOT) as MIPs potentially selective for immunoglobulin G (IgG) used as a template protein. Docking was combined with quantum chemical calculations. The protein was first docked by Glide software [90,91] to predict the most favorable binding poses on a monomer as well as the arrangements of multiple monomers around the protein, then the poses were refined based on a quantum-mechanically polarized ligand workflow to estimate the cumulative strength of H-bond interactions. The quantum chemical calculations were performed in Gaussian 09 at the hybrid DFT B3LYP/6-31G level. Results revealed the strength of H bond interaction between different monomeric substrates of MIP and template proteins in the pre-polymerization process, whereas docking provided the most favorable binding poses of protein over functional monomers with non-covalent binding energy. Computational predictions indicated that among other functional monomers mPD based MIP could be the optimal choice for the sensitive detection of IgG. Results were validated by experimental data.

In one study [92] by Zanuy et al., a thin film of Poly (hydroxymethyl-3,4-ethylendioxythiophene) (PHMeDOT) on a steel substrate was examined by classical MD for its ability to detect glucose and fructose. MD simulation results suggested that the system could be used as a sensitive tool for sugar monitoring of diabetic patients due to its specific hydrogen-bonding interactions with glucose with respect to other sugars, e.g., fructose. RESP charges and the amber force field [93] were used in NAMD [79]. Radial distribution function and residence time were computed to differentiate between the surface affinity towards glucose and fructose over thin film.

To summarize, QM calculations demonstrated to be a suitable tool to obtain the optimized structure of conducting polymer grafting on surfaces and/or to understand the adsorption energy between template and MIP, on the basis of both the covalent interactions and non-covalent interactions, i.e., hydrogen-bonding. QM can be combined with classical atomistic simulations to understand the changes in the conformation of polymers on a longer time scale, to estimate the surface/analyte noncovalent interaction, and to obtain the proper protein–surface binding orientation.

### 3.1. Aptasensors

Aptamers are single-stranded oligonucleotides (~100 bases) analogs of antibodies with high affinity to specific targets, such as cells, viruses, proteins, inorganic materials, and coenzymes [29,30,31]. Several studies have been devoted to the development of biosensors using aptamers as bioreceptors, namely, the biosensors can be based on various nanomaterials, such as quantum dots, metal nanoparticles (NP), and single- and multi-walled CNT [94]. Computational methods have significantly expanded the possibilities of aptamer design for biosensing applications. A full set of in silico methods have been applied [94], such as docking, molecular dynamics (MD), and statistical analysis. A typical modeling workflow consists of (i) aptamer structure prediction, (ii) docking between target and aptamer, (iii) MD simulations to determine the stability of aptamer/ligand complexes and the relative binding energies at a higher resolution. Additionally, simulations of aptasensors have involved the (iv) aptamer-functionalized nanomaterials interacting with the ligand. In some cases, the whole computational procedure is iteratively repeated. Thus, docking and MD simulations have been assessed as complementary tools to augment experimental studies on the interaction between aptamers and their ligands opening up new pathways for biosensing design.

As reported by Buglak et al. [94], not only are docking, molecular dynamics, and quantum-chemical calculations employed for in silico design of aptasensors but also QM/MM methods are used to predict the structural patterns of the aptamer/ligand complex [95], whereas QSAR [96] and machine learning [97], are envisaged to provide important advancements in the field of aptamer’s design and modeling in the near future.

Single-stranded RNA aptamers are emerging as important novel diagnostic tools, for their easiness of fabrication, for their ability to rapidly detect target molecules, and their reusability [98]. The aptamers within biosensors are immobilized onto the transducer surface to maximize their access to target molecules but as a potential drawback, the immobilization can lead to a loss of affinity. However, a clear understanding of the immobilization process at the molecular level is difficult to tackle with experiments alone.

**Figure 4:**
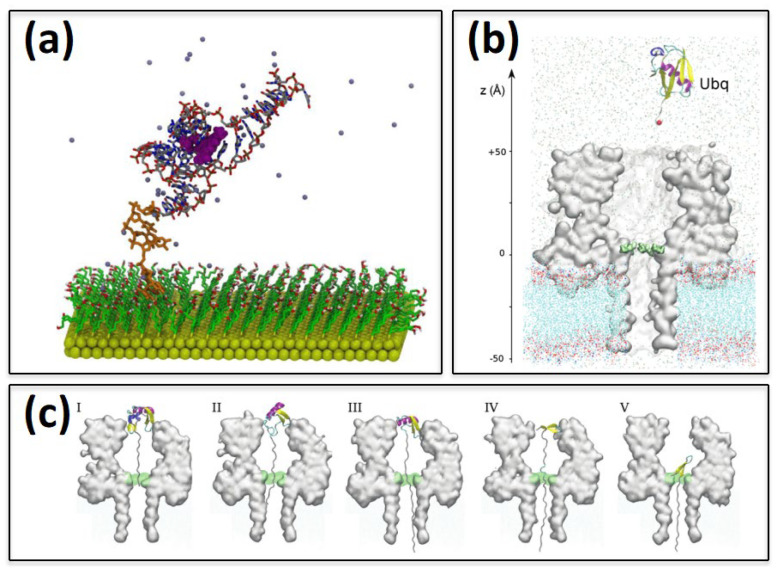
(**a**) Thymidine (T6) spacer attached with RNA/Flavin immobilized on a hexanol-thiol-functionalized gold surface in aptasensor simulation studies. Reprinted with permission from Ref. [99]. Copyright© 2022, American Chemical Society. (**b**,**c**) Translocational pathway of ubiquitin inside α-hemolysin (αHL) inserted in POPC bilayer in a nanopore-based sensor, (**b**) ubiquitin is pulled in a steered MD by N terminal inside the nanopore and (**c**) from the same C terminal, as obtained from Reference [101] and reproduced with permission from the Copyright© 2022 Royal Society of Chemistry.

In this respect, a recent advancement has been provided by a computational study [99] of the entire Flavin mononucleotide RNA aptamer biosensor system for which experimental data were not available. The Flavin receptor was simulated in complex with its corresponding RNA aptamer, anchored with a six thymidine (T6) spacer, and immobilized on a hexanol–thiol-functionalized gold surface. The PDB structure RNA/Flavin complex was simulated (Figure 4a) through classical all-atom MD using [99] ff99SB-ILDN and GAFF parameters, using the AM1-BCC method to incorporate partial charges within the Amber 12 software [100]. The hexanol–thiol modified Au (111) surface was optimized using DFT, then the T6-RNA/Flavin complex was grafted onto the surface. Remarkably, through MD computations, the Flavin binding free energy and the role of the aptamer’s orientation and location on the surface as a function of the linker were estimated, being a keystone for the recognition.

During the simulations of T6-RNA/Flavin tethered on hexanol–thiol functionalized gold surface, the aptamer was found to remain parallel to the surface, separated at ~36 Å, ignoring surface effects potentially leading to a loss of affinity. Instead, a structural reorganization of the Flavin aptamer binding mode was observed which could be related to a loss of affinity and that was induced by an anisotropic distribution of sodium cationic densities, as manifested by the radial distribution function of the monovalent Na^+^ at the interface between the surface and the aptamer. Overall, results assessed the capability of atomistic simulations to examine the potential role of the interfacial ions distribution and to suggest how to tune it along with the relative orientation of the spacers group anchoring the aptamers, in order to design an aptasensor with higher performances in terms of selectivity and efficiency.

In another study [102], Rhinehardt et al. focused on the design of an aptasensor based on an aptamer (anti-MUC1) acting as a bioreceptor for the breast cancer biomarker mucin 1 (MUC1) protein, by means of extensive MD simulation. Since the MUC1 protein sequence contained several peptide variants targeted by the aptamer, multiple MUC1 and MUC1-G peptide-binding combinations were simulated using GROMACS [61] and Amber99sb [56] force field. Different orientations and binding patches could be monitored by calculating the center-to-center distance between the peptide and the aptamer molecules. The radius of gyration, the ion distribution around aptamers, and the hydrogen bond analysis provided evidence that even small differences in the peptide sequence could significantly affect the binding complexes. Simulations assisted the disclosure of the aptamer’s orientations and locations on the surface which were critical parameters in order to further enhance the biosensor detection mechanisms.

Phanchai et al. reported [103] a MD-based study on the detection of ochratoxin A (OTA), a type of mycotoxin with a colorimetric aptasensor based on the assembly of salt-induced gold nanoparticles (AuNPs). In particular, the molecular recognition of the OTA-aptamer was investigated at the atomistic level using a hybrid solvation model. The structure of OTA was first optimized with the Gaussian09 [73] software package at the HF/631G* level, and partial charges were derived from ANTECHAMBER [93]. Then, the system which was composed of a ssDNA aptamer, target molecules, and citrate-capped AuNPs, was simulated with a hybrid model of the solvent in which the bulk area was represented by a coarse-grained (CG) solvent model, called WatFour (WT4), whereas the water around the solute was considered in atomistic details (TIP3P). Binding residues were marked through a distance cut-off and hydrogen bond formation. The potential role of divalent ions, such as Mg^2+^, in the bridging interaction, was proved to be significant. Additionally, the binding modes on citrate-capped gold nanoparticles (AuNP) were elucidated through quantum calculation. The underlying mechanism behind adsorption of the aptamers on the negatively charged citrated AuNP (electrostatic nature) in the absence and the presence of the OTA molecule, provided atomistic insight on the molecular mechanisms of the AuNP-based aptasensor, which is a fundamental requirement for future developments of novel sensors detecting small molecules.

### 3.2. Nanopore Based Biosensors

Nanopores are ~100 nm-sized devices typically consisting of a charged biomolecule (protein/DNA) guided in a lipid membrane in the presence of an electric field and ionic solution. They can be used for single-molecule tracking, particularly for the detection of any change in a desired protein’s concentration, size, shape, conformation, sequence, or binding event with ligands in solution. Nanopores also provide information on reaction kinetics, diffusivity, post-translational modification, unfolding phenomenon, and more [32,33], on the basis of an output ionic response or “pulse”. Moreover, the surface of a nanopore can be functionalized with suitable recognition elements to bind with the target sequence. However, the detection of any biomolecular process within the nanopore can be challenging due to the possibility of the protein undergoing transient or permanent conformational changes, associated with the protein placed within the tunnel or an improper binding of the protein with the suitable ligand inside the pore. Moreover, a lack of sufficient residence time of the protein within the nanopore could become an obstacle in the detection of the response signal. Due to the presence of non-specific bindings over the pore walls and the complexity of the length and time scale (~µs) involved, the overall sensing mechanism is difficult to be addressed from a solely experimental perspective, thus computational studies can be considered a complementary tool.

A computational study [101] was reported to understand the translocational pathway of ubiquitin inside α-hemolysin (αHL)-based nanopores (Figure 4b,c) through all-atom MD simulation. The rationale was to characterize the coupling between protein translocation and unfolding, namely the co-translocation, which is one of the open issues in narrow nanopores. Rearrangements of the protein secondary structure elements were observed in robust translocation intermediates. The proteins and the POPC bilayer were simulated following the standard protocol using the NAMD [79] and CHAMM36 force fields [41], whereas the unfolding pathway of ubiquitin inside the pore was investigated using a steered MD simulation. In particular, backward burial analysis, namely a measure of the interaction of a residue with the untranslocated portion of the proteins (assumed to be in a folded state), was used to locate the number of long-range contacts between intraresidues of the native protein throughout the entire steered dynamics. The simulated data provided evidence that the native protein structure was more important than the specific pore–protein interaction in controlling the co-translocation unfolding pathway. However, the complexity of the involved process had been identified as a limit in the actual feasibility of the protein sensing.

The above atomistic results [101] were confirmed by coarse-grained simulations based on the Gō model [104], where each monomer of ubiquitin was represented at a lower resolution by lattice beads connected by a lattice constant, having local as well as non-local contact interactions. The protein αHL was addressed via two coaxial confining cylinders, while the number of residues of ubiquitin at the bottleneck of the inner cylindrical pore and their residence time were monitored. Another important observation was the detection of the N terminal β hairpin region of ubiquitin enhancing the constriction of αHL. Interestingly, this N terminal β hairpin region of ubiquitin was targeted for several post-translational modifications (PTMs) processes. Thus, a new strategy to exploit the co-translocational pathway of a protein through nanopore-based sensors in detecting PTMs was assessed.

Ramachandran et al. reported [105] the dynamics of DNA translocation along with DNA sequencing mechanism through differently functionalized (hairpin/HPL and ssDNA) silicon-based nanopore using coarse-grained MD simulation in GROMACS [61]. DNA bases and the backbone groups of DNA were represented as two separate interaction sites, bonded interactions, such as bond stretching, bending, torsion, and the non-bonded terms for base stacking, the effect of excluded volume, hydrogen bonding, and electrostatic interactions, were included. The interactions between nanopore and DNA were described by LJ potentials, whose parameters were extracted from all-atom MD simulations. The translocational velocity of target DNA was analyzed under different conditions, pointing to a potential role of flexibility, orientation, density, and chemical composition of the surface tethered groups of the nanopore. Effective pore diameter and the biasing voltage in optimizing selectivity and sensitivity of this type of solid-state nanopore-based sensor were addressed, and it was observed that the target DNA translocated was faster through ssDNA tethered nanopores than HPL modified ones. Thus, the flexibility and orientation of tethered molecules could significantly affect the translocation dynamics of DNA. This study emphasized a novel design of DNA transporter for the rapid and desired diagnostics.

## 4. Conclusions

We reviewed in silico studies on functionalized nanomaterials for promising applications in biosensing. We included several classes of nanomaterials having proteins as targets, namely graphene-based biosensors, self-assembled monolayer-based biosensors, polymer-based biosensor, aptasensor, nanopore-based biosensor. A full set of molecular modeling methods have been reported, spanning from Brownian Dynamics, Molecular Dynamics, Quantum-Chemical calculations, and more recently Coarse-Grained and Machine Learning techniques.

Many evidences have been collected showing that a deeper insight into interfacial interactions occurring in biosensors can be successfully gained with a balanced combination of in vitro experiments and computer simulations. Simulations combine sampling and force field. Sampling techniques must be chosen depending on the system to investigate but plain MD has proven to be the most reliable approach in the large majority of reported studies, enabling direct observation of the sequence of events, including transition states and the structure and dynamic behavior of water, ions, and other solvent components.

In order to perform modeling and to correctly describe the protein/nanomaterial interfaces, several developments of atomistic potentials for the novel functionalized nanomaterials acting as biosensors, have been reported mainly from ab initio calculations. Investigations systematically comparing different force fields would be useful.

Moreover, it should be stressed that each of the mentioned methods can reveal one or more different aspects relevant to the sensing process. For example, Brownian Dynamics provides orientations and interaction sites of the initial binding, Molecular Dynamics refines such findings and also provides conformational rearrangements within the sensing layers and the biomolecules. Quantum-Chemical calculations can provide estimates of the effect of binding on electronic structures and optics, as well as of binding energy. More specifically, hybrid approaches, such as QM/MM, can be conveniently applied to bridge typical time and length scales of novel aptamers-based biosensors [94,95]. Coarse-grained models also have great potential as they may reveal structural changes and processes on time scales inaccessible to MD. However, building coarse-grained models tailored for the target systems requires an initial effort that has limited their use in this field so far.

The notorious limitations related to the computational cost of QM simulations and limitations due to the lack of dispersion interactions can be improved by future methodological developments, but we can envisage that approaches other than DFT, such as Quantum Monte Carlo, may also become popular for investigating interfacial interactions of biosensors due to the constantly growing size of supercomputers. Thus, QM approaches are still playing an important role and they can be used either to provide a detailed picture of the local interfacial interactions or as a basis for developing classical atomistic models. To conclude, capturing the time and length scales of a complete biosensing mechanism process overall requires employing multi-scale approaches, including not only atomistic but also mesoscopic and coarse-grained models.

To date, in silico predictions and the related computational analysis have been confirmed by in vitro experiments, whenever available, assessing their effectiveness. The in silico predictions have been confirmed in vitro and have contributed to augmenting the affinity of functionalized nanomaterials with respect to their target analytes (although computational predictions of LODs seem still out of reach), along with ensuring the stabilization of the required conformations. In the framework of further work, we envision that this review will support further developments in this field to improve the recognition of biological systems with high selectivity. Further, it highlights areas in need of future emphasis.

## Data Availability

Not applicable.
